# Comparative effectiveness of hyaluronic acid, platelet-rich plasma, and platelet-rich fibrin in treating temporomandibular disorders: a systematic review and network meta-analysis

**DOI:** 10.1186/s13005-023-00369-y

**Published:** 2023-08-26

**Authors:** Jingjing Xu, Hui Ren, Shuwei Zhao, Qian Li, Ce Li, Guangjie Bao, Hong Kang

**Affiliations:** 1 Key Laboratory of Stomatology of State Ethnic Affairs Commission, Northwest Minzu University, Lanzhou, 730030 China; 2 Department of Temporomandibular Joint and Occlusion, School/Hospital of Stomatology, Lanzhou University, Lanzhou, 730000 China

**Keywords:** Temporomandibular disorder, Arthrocentesis, Hyaluronic acid, Platelet-rich plasma, Platelet-rich fibrin, Network meta-analysis

## Abstract

**Objective:**

This study aims to compare the efficacy of intra-articular injections of hyaluronic acid (HA), platelet-rich plasma (PRP), and platelet-rich fibrin (PRF) for treating temporomandibular disorders (TMDs) and summarize their mechanisms of action.

**Methods:**

Randomized controlled trials (RCTs) published until November 13, 2021, were identified using electronic and manual searches. Each study was evaluated for the risk of bias using the Cochrane risk of bias tool. The studies found via searches were categorized by follow-up time (1, 3, or 6 months). Evidence quality was graded according to the GRADE system.

**Results:**

Twelve RCTs were included that involved 421 patients with TMD. The network meta-analysis showed that all treatment groups improved compared to the placebo groups in terms of pain and maximal mouth opening (MMO). For pain evaluated via the visual analog scale, PRF exhibited better analgesic effects than PRP or HA after 1 and 3 months. PRP appeared to be more effective than PRF was after 6 months but there were no statistically significant differences between the two. For MMO, the effect of PRP was superior to those of PRF and HA after 1 month. However, after 3 and 6 months, PRF provided more encouraging results in improving MMO.

**Conclusion:**

PRP and PRF exhibited similar short-term efficacy in treating TMD, while PRF was more advantageous in terms of long-term efficacy. Therefore, PRF was recommended for treating TMD.

**Supplementary Information:**

The online version contains supplementary material available at 10.1186/s13005-023-00369-y.

## Introduction

Temporomandibular disorders (TMDs) are an umbrella term for musculoskeletal disorders referring to pain and/or dysfunction of masticatory muscles, temporomandibular joints (TMJ), and associated structures [1–3]. TMD is indicated to be a multifactorial disorder, which agrees with the biopsychosocial model of illness [4]. Pain is an essential factor affecting patient quality of life, and the main driving factor for patients with TMD to seek treatment [5, 6]. Treatment for TMD aims to reduce pain, restore normal mandibular movements, and enhance quality of life. Different therapies, including conservative treatment, minimally invasive surgical operations, and invasive surgical operations, have been widely tested to treat TMD. Conventional treatment methods, such as medication, physiotherapy, and occlusal splint, are often the first treatment option recommended in the early stage of the disease because they are almost noninvasive and can treat mild-to-moderate TMD [7]. With disease development, joint degeneration or osteoarthritis will occur. When simple conservative treatment is ineffective, minimally invasive surgical operations can be used, such as arthrocentesis and intra-articular injection of agents; however, caution is required for surgical operations that may cause significant damage to patients and is usually considered to be the last choice of action [8–11].

TMJ arthrocentesis was first described in 1991 by Nitzan et al. [12]. Arthrocentesis usually involves the use of normal saline or Ringer’s solution to irrigate and expand the upper joint space under local anesthesia to remove inflammatory mediators, alleviate pain, disrupt any adhesion, release the articular disc, and improve joint mobility [9]. Hyaluronic acid (HA) has been clinically used as a common agent for intra-articular injections, whereas platelet-rich plasma (PRP) and platelet-rich fibrin (PRF) are new injectable agents that have been used more recently. PRP and PRF are platelet concentrates obtained via centrifugation of the autologous blood of patients and are considered to have an excellent therapeutic effect on patients with TMD [13]. Only a few systematic reviews and network meta-analyses that probed the efficacy of HA and PRP in treating TMD have been reported [10, 14–24], and a comparison of the efficacy of HA, PRP, and PRF in treating TMD has not yet been reported. Therefore, a network meta-analysis is required to compare and rank the efficacy of HA, PRP, and PRF in treating TMD to provide clinicians with recommendations when selecting treatment options for TMD.

## Materials and methods

### Protocol and registration

This systematic review was registered with PROSPERO under the registration number CRD42022303863.

### Eligibility criteria

Following the population, intervention, comparison, outcomes and study (PICOS) principles, the following inclusion criteria were employed: (P) Patients diagnosed with TMD; (I) Intervention: Intra-articular injections of HA, PRP, or PRF with/without arthrocentesis in patients diagnosed with TMD; (C) Comparator: Patients receiving arthrocentesis alone, including lysis and lavage using normal saline or Ringer’s solution without injection of any medications (placebo group); (O) Outcomes, primary outcome: pain, and secondary outcome: MMO; (S) Study design: RCTs (initial evidence hierarchy for RCTs was rated “high” [25]).

Criteria for excluding articles were: (1) Studies where participants had a history of severe systemic diseases or were taking medications that might have affected the assessment; (2) review articles and animal studies; (3) studies involving participants treated with other treatments that may have influenced the assessment; (4) studies assessed as high risk by the Cochrane risk of bias tool; and (5) studies reported in languages other than English.

### Research strategy

An electronic search was conducted in PubMed, Cochrane Library, Embase, Web of Science, and Scopus using MeSH terms and keywords to find relevant studies published until November 13, 2021; moreover, manual reviewing of the studies was conducted. The retrieval strategy is described in Additional file 1.

### Study selection and data extraction

Based on title and abstract reading, three reviewers (JX, CL, and SZ) independently excluded duplicates and irrelevant studies. Study selection was completed by reading the full text of the remaining literature. For the included studies, the Cochrane risk of bias tool (Review Manager 5.4) was used by two reviewers (HR and QL) to assess the risk of bias. Finally, the required data were independently extracted by reviewers (HK and GB) from the included studies. Extracted data covered the basic information available, characteristics of participants, and detailed information about the interventions and outcomes. Any dispute was decided through discussion.

### Outcome assessment

Pain was assessed via visual analog scale scores as the primary outcome, and MMO was assessed as the secondary outcome. Reviewers used the mean difference (MD) and corresponding 95% confidence interval (CI) to calculate the effect size.

### Network meta-analysis

The “mvmeta” and STATA 15.1 were used to perform statistical analyses. The network geometry emerged via drawing a plot to observe whether studies contained in various treatment schemes were connected [26]. The transitivity among studies was considered by assessing the similarity of PICOS in each study. The “design-by-treatment” interaction model was used to check the consistency of the entire network [27]. A statistical test of the entire network must be significant (*p* > 0.05) for the reviewer to accept the inconsistency. Then network and forest plots were prepared using the network commands. Furthermore, reviewers used the surface under the cumulative ranking (SUCRA) curve to analyze the treatment hierarchy and identify treatments that had superior interventions. The higher the SUCRA, the better the therapeutic effect of the intervention [28]. Afterwards, Egger and Begg tests were used to examine potential publication bias in addition to the funnel plots. Finally, to evaluate the evidence quality, the GRADE system was adopted according to the results of the above network meta-analysis [29].

## Results

### Study selection

**Fig. 1:**
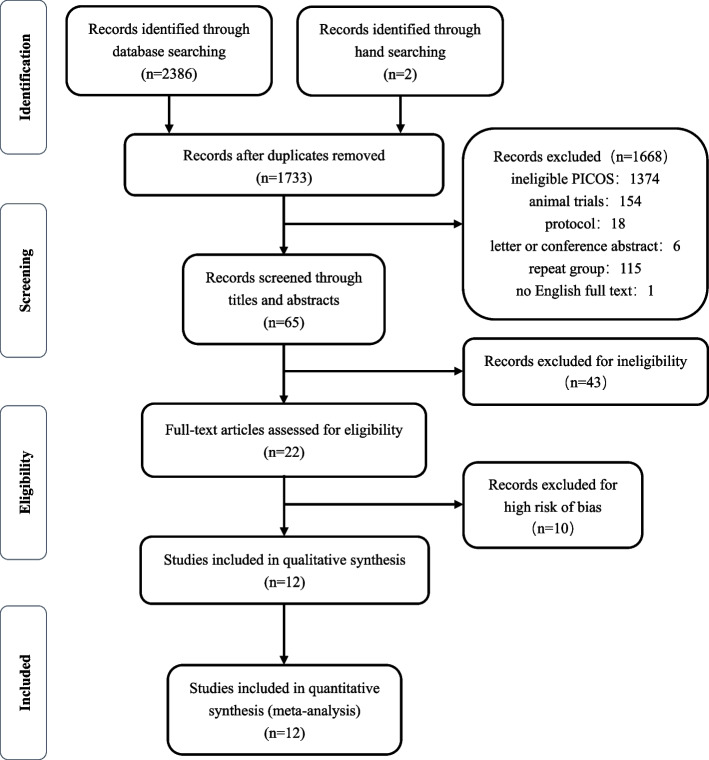
Flowchart of the selection process

A total of 2,388 potentially relevant studies were retrieved, of which 655 were excluded because of duplication. The titles and abstracts of the remaining 1,733 studies were screened in detail, and 65 studies that could meet the inclusion criteria were obtained. These 65 studies were carefully full-text screened, and the potential eligibility of 22 studies among the 65 studies was determined [30–51]. After further screening, 12 studies were finally included for network meta-analysis [34, 36, 38, 39, 41, 42, 45–47, 49–51]. Figure 1 shows the inclusion and exclusion processes.

### Characteristics of the included studies

In total, 12 RCTs were included in the network meta-analysis of 421 patients with TMD, involving 92, 82, 38, 187, and 22 patients in the HA, PRP, PRF, placebo, and other groups, respectively. Except for those not reported in the literature, the mean age range of the patients was 22.17 ± 3.61 to 51.50 ± 12.80 years. The search results included 12 direct comparisons: four were HA versus placebo, four were PRP versus placebo, two were PRF versus placebo, and two were HA versus PRP versus placebo. The characteristics of the 12 included RCTs are summarized in Table 1.

**Table 1:** Main characteristics of included studies

	**Study**	**Country**	**Participants**					**Intervention**		
			**Group**	**Male/Female**	**Age (years)**	**N**	**TMD diagnosis**	**Type**	**Centrifugal method**	**Centrifugal speed/time**
HA:PO	1 Gorrela, H/2017	India	HA	34/28	no report	62	ID	arthrocentesis + intra-articular injection 1 ml HA	—	—
			placebo					arthrocentesis	—	—
	2 Patel, P/2016	India	placebo	9/21	no report	30	ID	arthrocentesis	—	—
			HA					arthrocentesis + intra-articular injection 1 ml HA	—	—
	3 Rao, J. K. D/2019	India	placebo	7/3	37 ± 16.11	20	ID	arthrocentesis	—	—
			HA group	7/3	34.2 ± 12.54			arthrocentesis + intra-articular injection 1 ml HA	—	—
	4 Yapıcı-Yavuz, G/2018	Turkey	group 1	6/38	no report	44	DDwoR	arthrocentesis	—	—
			group 2					arthrocentesis + intra-articular injection HA	—	—
			group 3					arthrocentesis + intra-articular injection methylprednisolone acetate	—	—
			group 4					arthrocentesis + intra-articular injection tenoxicam	—	—
PRP:PO	5 Chandra, L/2021	India	PRP	no report	32.3 ± 10.4	44	TMD	intra-articular injection 0.6 ml PRP	double centrifugation	1800 rpm 15 min; 3500rpm10min
			placebo		29.00 ± 8.3			arthrocentesis	—	—
	6 Hancı, M/2015	Turkey	PRP	no report	27.2 ± 13.4	20	DDwR	intra-articular injection 0.6 ml PRP	double centrifugation	1000xg 20 min; 1500xg 10 min
			placebo	3/7	25.4 ± 1.7			arthrocentesis	—	—
	7 Rajput, A/2020	India	placebo	7/5	22.17 ± 3.61	24	TMD	arthrocentesis	—	—
			PRP	2/10	27.83 ± 11.71			intra-articular injection PRP	double centrifugation	1800 rpm 15 min; 3500 rpm 10 min
	8 Singh, A. K/2021	India	PRP	4/8	34.75 ± 10.83	24	ID	arthrocentesis + intra-articular injection 1mlPRP	single-step centrifugation	2000 rpm 8 min
			placebo	2/10	36.41 ± 11.15			arthrocentesis	—	—
PRF:PO	9 Ghoneim, N. I/2021	Egypt	placebo	7/13	28.60 ± 8.42	40	ID	arthrocentesis	—	—
			PRF	4/16	26.45 ± 8.21			arthrocentesis + intra-articular injection 1.5 ml PRF	single-step centrifugation	700 rpm 3 min
	10 Karadayi, U/2021	Turkey	placebo	9/9	39.67	36	ID	arthrocentesis	—	—
			PRF	8/10	39.97			arthrocentesis + intra-articular injection 2 ml PRF	single-step centrifugation	700 rpm 3 min
HA:PRP:PO	11 Jacob, S. M/2021	India	PRP	4/12	40.56 ± 9.72	47	ID	arthrocentesis + intra-articular injection 1 ml PRP	single-step centrifugation	no report
			HA	6/9	46.53 ± 19.15			arthrocentesis + intra-articular injection 1 ml HA	—	—
			placebo	7/9	51.50 ± 12.80			arthrocentesis	—	—
	12 Toameh, M. H/2019	Syria	placebo	2/8	40.53	30	DDwoR	arthrocentesis	—	—
			HA	3/7	38.26			arthrocentesis + intra-articular injection 1 ml HA	—	—
			PRP	1/9	37.82			arthrocentesis + intra-articular injection 1 ml PRP	single-step centrifugation	3400 rpm 4 min

*HA* Hyaluronic acid, *PRP* Platelet-rich plasma, *PRF* Platelet-rich fibrin, *PO* Placebo, *ID* Internal derangements, *DDwR* Disc displacement with reduction, *DDwoR* Disc displacement without reduction, *TMD* Temporomandibular disorders

### Risk of bias assessment

**Fig. 2:**
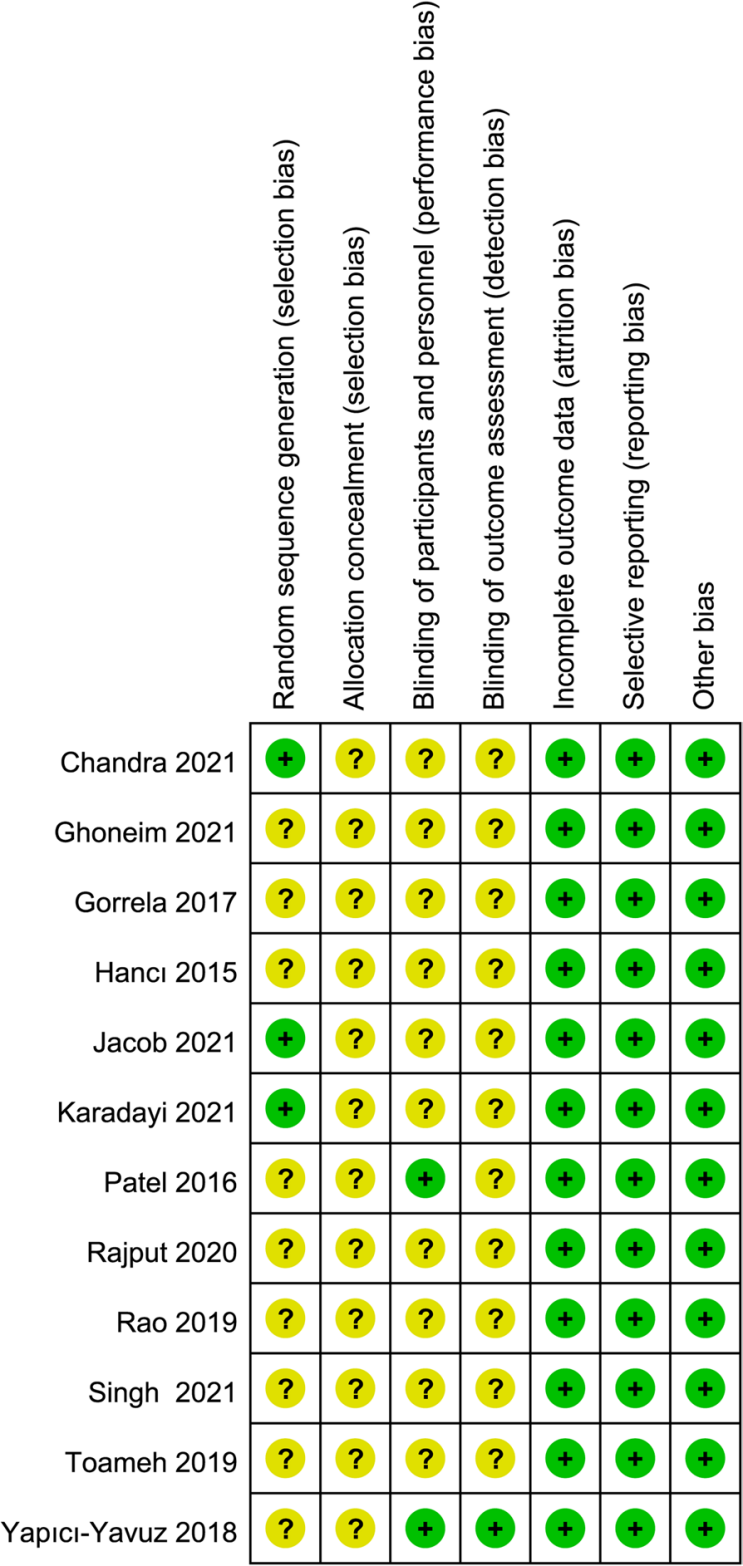
Summary of risk of bias of all included studies

The risk of bias for each included study is shown in Fig. 2. Three studies completely generated random sequences through random number lists, computer programs, flipping coins, and drawing lots, etc., and consequently they were considered to have a low bias risk in the random field [34, 41, 42]. No studies reported details on how allocation hiding was implemented. Two studies considered had a low bias risk in blinding of participants and personnel assessment [45, 51]. If it was not indicated whether the studies were blinded it would be considered as an unclear bias risk. Regarding detection bias, most studies were deemed to have unclear risk of bias [34, 36, 38, 39, 41, 42, 45–47, 49, 50]. Data losses were absent in all studies, and we consequently considered them to be at low risk of bias against incomplete outcome data. In addition, because prespecified results from the studies were reported and no other essential bias issues were identified, all included studies were considered to be at low risk for reporting bias and other bias.

### Results of the network meta-analysis

**Fig. 3:**
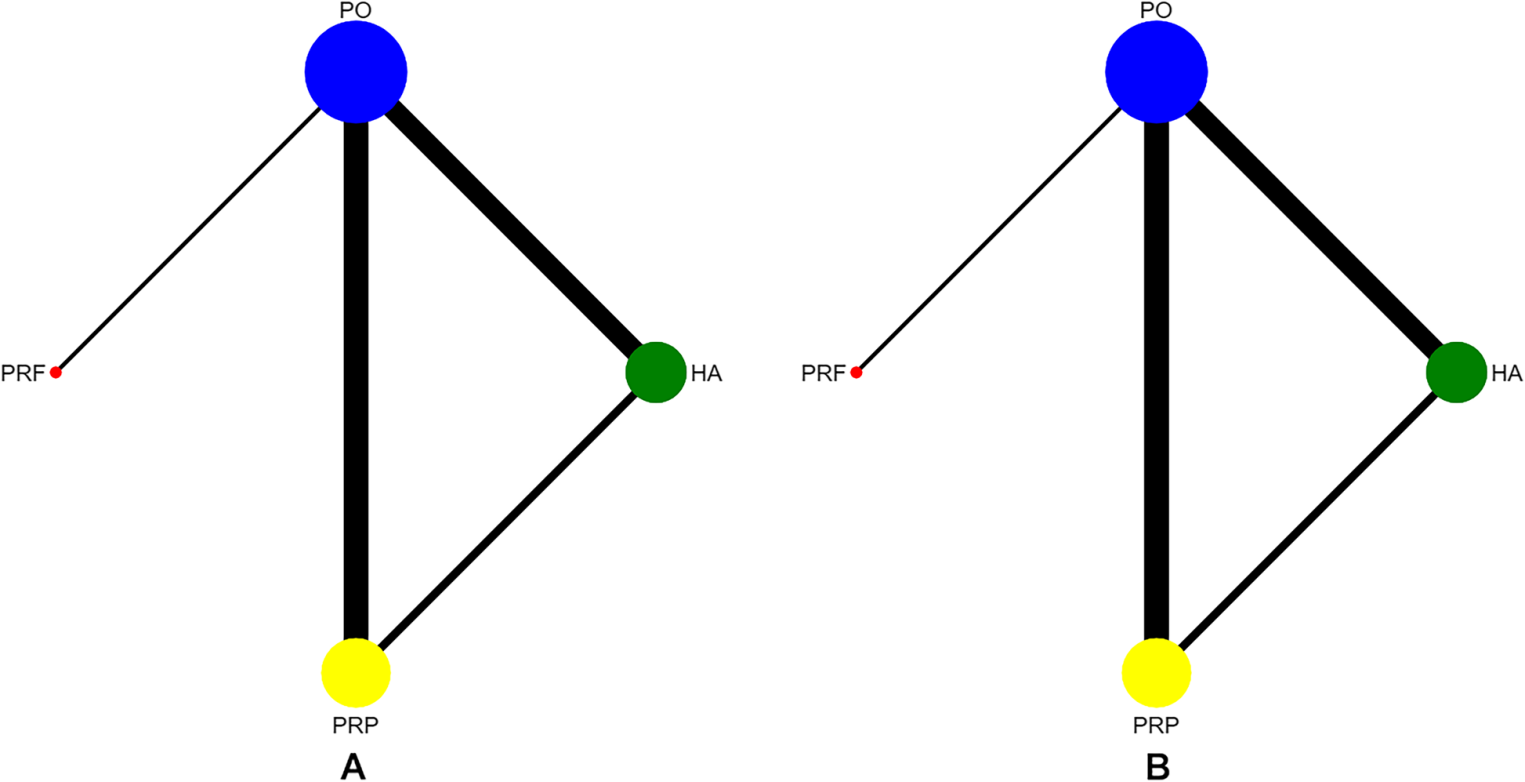
Network plot. **A** Pain after six months of treatment; **B** MMO after six months of treatment. HA: hyaluronic acid; PRP: platelet-rich plasma; PRF: platelet-rich fibrin; PO: placebo; MMO: maximal mouth opening

The network diagrams are shown in Fig. 3. Nodes with larger sizes have larger samples. A direct comparison between the two interventions is represented by the connection line between the two nodes; a thicker connection line indicates more abundant literature. The use of network meta-analyses indirectly allows the comparison of unrelated interventions. The transitivity among studies was determined by the reviewers after considering the similarity of PICOS in each study. The overall consistency test results of three follow-up periods of treatment (1, 3, and 6 months) were* p* = 0.008, 0.059, and 0.829 for Pain and *p* = 0.032, 0.045, and 0.07 for MMO, respectively. Studies involving inconsistencies in network results would be degraded in the assessment of evidence.

The results indicated that the pain intensity of PRP and PRF was significantly lower than that of placebo during the 1-month follow-up, and the analgesic effect of PRF was better than that of PRP (MD =  − 2.89, 95% CI: − 4.63 to − 1.15; MD =  − 1.04, 95% CI: − 2.00 to − 0.09; and MD = 1.49, 95% CI: − 0.94 to 3.92, respectively). The efficacy of HA was lower than that of a placebo but not significantly so (MD = 1.74, 95% CI: − 0.19 to 3.68). During the 3-month follow-up although the analgesic effect of PRF was the best, this was not significant. The comparison between PRF and placebo was significant (MD =  − 2.26, 95% CI: − 4.14 to − 0.37). During the 6-month follow-up, the analgesic effect of PRP appeared to be the most effective but was only significantly different compared with the effect of placebo (MD =  − 1.17, 95% CI: − 1.82 to − 0.51).

Although PRP showed a superior therapeutic effect for MMO after a 1-month follow-up, this remained insignificant. During the 3-month follow-up, the effect of PRF was more effective than that of HA in treating MMO (MD = 6.69, 95% CI: 2.11 to 11.28), and PRP was more effective than that of placebo (MD = 8.31, 95% CI: 4.81 to 11.82). PRF was associated with a superior treatment effect compared with that of PRP, although this was not significantly different (MD =  − 2.31, 95% CI: − 8.69 to 4.06). During the 6-month follow-up, the effect of PRF was significantly more effective than that of PRP, HA, and placebo (MD =  − 11.01, 95% CI: − 16.17 to − 5.86; MD = 8.72, 95% CI: 3.64 to 13.80; and MD = 11.12, 95% CI: 6.45 to 15.79, respectively). Table 2 shows the results of each group, and Additional file 2 presents the results of the forest plot.

**Table 2:** The meta-analysis results and corresponding qualities of evidence (GRADE)

One month after treatment			
PRP	^b^1.80(-6.68,10.28)/m	^b^5.49(-1.36,12.34)/m	^b^4.50(-1.88,10.88)/m
^a^1.49(-0.94,3.92)/m	PRF	^b^3.69(-3.81,11.19)/m	^b^0.81(-3.14,4.77)/m
^a^-0.36(-2.31,1.60)/l	^a^-1.85(-3.83,0.13)/l	HA	^b^2.99(-2.78,8.76)/l
^a*^-2.89(-4.63,-1.15)/l	^a*^-1.04(-2.00,-0.09)/m	^a^1.74(-0.19,3.68)/m	PO
Three months after treatment			
PRP	^b^-2.31(-8.69,4.06)/l	^b^4.38(-1.70,10.47)/m	^b*^8.31(4.81,11.82)/m
^a^1.38(-0.76,3.52)/m	PRF	^b*^6.69(2.11,11.28)/m	^b^1.62(-1.33,4.57)/m
^a^-0.47(-1.85,0.91)/l	^a^-1.85(-4.02,0.31)/l	HA	^b^1.38(-3.43,6.20)/l
^a^-0.88(-1.89,0.14)/m	^a*^-2.26(-4.14,-0.37)/m	^a^-0.40(-1.50,0.70)/m	PO
Six months after treatment			
PRP	^b*^-11.01(-16.17,-5.86)/l	^b^-2.29(-4.85,0.27)/l	^b^0.11(-2.07,2.29)/l
^a^-0.17(-2.88,2.54)/m	PRF	^b*^8.72(3.64,13.80)/m	^b*^11.12(6.45,15.79)/m
^a^-0.79(-1.65,0.07)/m	^a^-0.62(-3.33,2.09)/m	HA	^b*^2.40(0.40,4.40)/m
^a*^-1.17(-1.82,-0.51)/m	^a^-1.00(-3.63,1.63)/m	^a^-0.38(-1.06,0.30)/m	PO

*PRP* Platelet-rich plasma, *PRF* Platelet-rich fibrin, *HA* Hyaluronic acid, *PO* Placebo, *H* High-quality evidence, *M* Moderate-quality evidence, *L* Low-quality evidence, *VL* Very Low-quality evidence

Interventions: PRP, PRF, HA, PO

^a^VAS difference value around treatment

^b^MMO difference value around treatment *; blod: statistically significant

### Rank probability

**Fig. 4:**
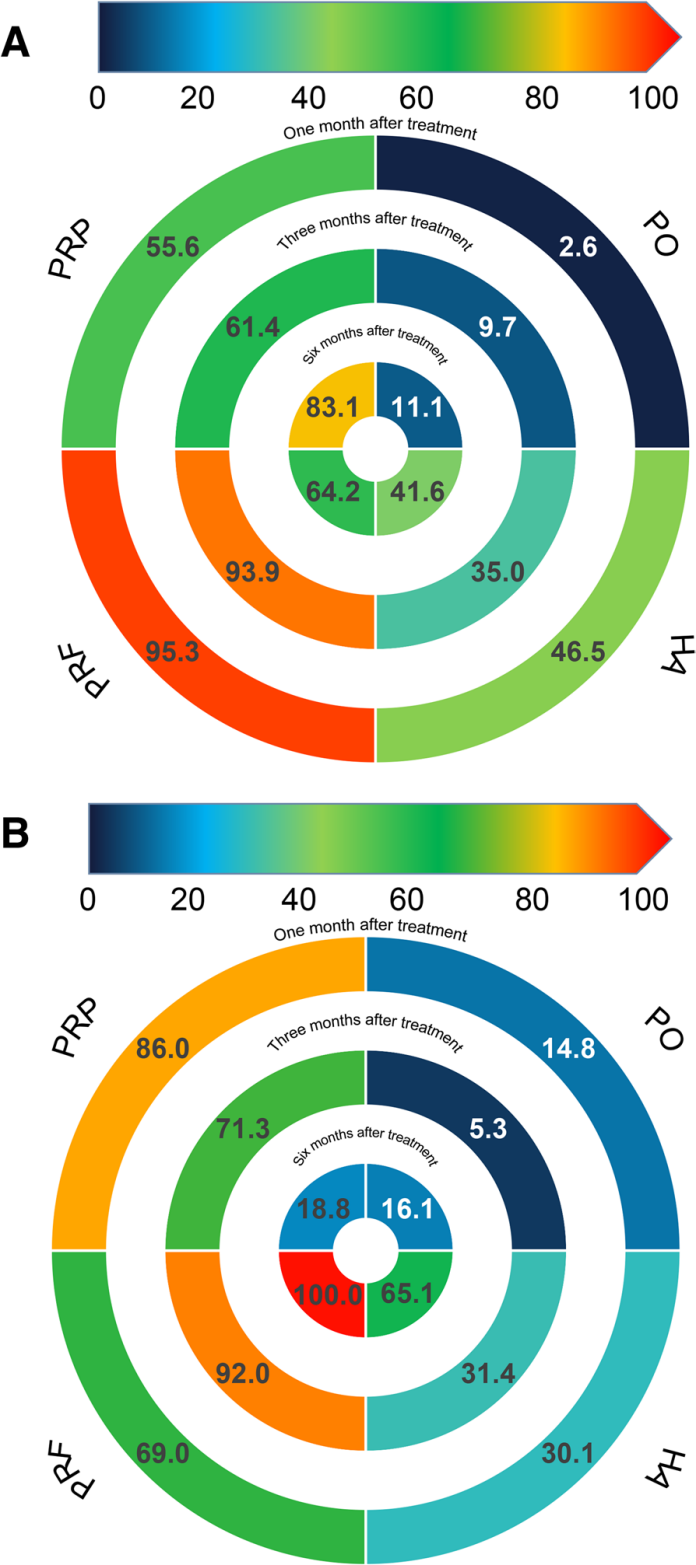
Rank-heat plot. **A** Ranking likelihood of pain relief after treatment for all interventions; **B** Ranking likelihood of MMO improvement after treatment for all interventions. HA: hyaluronic acid; PRP: platelet-rich plasma; PRF: platelet-rich fibrin; PO: placebo; MMO: maximal mouth opening

Figure 4 shows the ranking probability of the efficacy of different interventions at the three follow-up periods. The effects of pain relief and MMO improvement in patients with TMD were ranked by different interventions within three follow-up periods. For pain, 1 and 3 months after treatment, PRF was the most likely to be the best intervention (95.3% and 93.9%, respectively). The possibility of PRP being the best intervention was 83.1% when received for 6 months. The most effective treatment for MMO was 1 month treatment with PRP, with a probability of 86.0%. After 3 and 6 months of treatment, the likelihood that PRF was the best intervention was much higher than that of other interventions (92.0% and 100.0%, respectively).

### Publication bias

The funnel plots of the three follow-up periods were symmetrical, which showed that the publication bias on the included studies was acceptable (Additional file 3). Moreover, Egger and Begg tests did not provide any evidence to support publication bias for the three periods after treatment (*p* > 0.05).

### Quality assessment

In the three follow-up over periods, the quality of evidence based on the GRADE system was assessed as high-, moderate-, low-, and very low-quality evidence. The quality of evidence was degraded in several comparisons through inconsistency or imprecision. Table 2 shows the quality assessment for all the comparisons of network meta-analysis.

## Discussion

In the meta-analysis, we analyzed the available clinical studies on HA, PRP, and PRF for treating pain and MMO in patients with TMD and showed that HA, PRP, and PRF were all more effective than placebo for treating these conditions in the patients. According to the SUCRA, PRF showed the most obvious pain relief after 1 and 3 months of treatment, followed by PRP. Although the efficacy of PRP was slightly higher than that of PRF after 6 months of treatment, the efficacy of PRF remained much higher than those of HA and placebo. For MMO, PRP seemed dominant after 1 month of treatment and after 3 and 6 months of treatment, PRF was ranked first with the highest SUCRA score. Thus, over a short-term, PRP and PRF have similar efficacy for treating patients with TMD, while PRF is more advantageous in terms of long-term efficacy. Therefore, we recommend PRF as the optimum intra-articular injection agent for TMD patients.

HA is a glycosaminoglycan polysaccharide secreted by type B synoviocytes and naturally occurs in cartilage and synovial fluid [32, 33, 40]. The essential role of HA is to nourish, lubricate, and stabilize the TMJ [52]. Studies have found that the molecular weight of HA in the synovial fluid of patients with TMD (internal derangements and osteoarthritis) is decrease [48, 53, 54], whereas intra-articular injection of HA could supplement the synovial fluid and promote the production of endogenous HA [55]. Quinn and Bazan [56] found prostaglandin E2 and leukotriene B4 in synovial fluid of pain and dysfunction in the TMJ and suggested that joint pain was related to the level of inflammatory chemical mediators. Gorrela et al. [38] considered that HA injection could remove inflammatory mediators in the TMJ. The analgesic effect of HA was found to be caused by the decreased sensitivity of stretch-activated channels to mechanical forces [57], and higher molecular weight HA could effectively block the pain response by reducing the mechanical sensitivity of these channels In comparison, lower molecular weight HA was not considered as effective as higher molecular weight HA in blocking this response [57]. In addition, the improvement of MMO in patients with TMD may be related to the lubrication and analgesic effect of HA [30]. Therefore, we consider that intra-articular injection of HA can directly and indirectly increase nourishment, lubrication, and maintenance of joint stability to repair the damaged cartilage. HA can also effectively prevent the release and spread of inflammatory mediators and relieve joint pain by reducing joint pain receptors. Several RCTs have demonstrated the effectiveness of HA in treating TMD to relieve pain and improve MMO [38, 45, 47, 51].

PRP is extracted from centrifuged blood samples and is a concentrate of platelets and related growth factors [17, 18, 58, 59]. The platelet concentration has been found to differ with different preparation schemes of PRP, and the hematologic variation of patients may affect the final preparation of PRP [60]. PRP usually contains 4–fivefold the average platelet concentration in whole blood. In 2001, Marx considered the platelet concentration in blood to be at least 1 × 10^6^ platelets per µL of plasma in PRP, making a quantitative index for a PRP standard [61]. The basic scientific principle of PRP therapy is that PRP initiates tissue repair of damaged sites by releasing multiple bioactive factors and adhesion proteins [62]. During tissue repair, various growth factors, cytokines, and locally acting regulators act via endocrine, paracrine, autocrine, and intracrine mechanisms [62]. The platelets in the newly prepared PRP are dormant and need to be activated, usually by thrombin and calcium, to release more mediators [63].

The underlying mechanism of PRP-based therapy for treating patients with TMD is unclear. However, PRP may help release anti-inflammatory cytokines to inhibit the inflammatory response by releasing various growth factors that promote chondrocyte proliferation and cartilage repair and stimulate the production of endogenous HA.

PRP can release various anti-inflammatory cytokines to inhibit the inflammatory response in various ways. Through this mechanism, PRP can block the biological activity and signal transduction of inflammatory factors, including soluble tumor necrosis factor-α (sTNF-α) receptor antagonist I (sTNF-RI), sTNF-RII, interleukin-1 (IL-1) receptor antagonist (IL-1ra), IL-4, IL-l0, IL-13, and interferon-γ (IFN-γ) [64]. In addition, PRP can exert anti-inflammatory effects by reducing the transactivation of nuclear factor kappa-B (NF-κB). The proinflammatory cytokines IL-1 and TNF-α can activate NF-κB, a critical regulatory factor in inflammation. Hepatocyte growth factor can reduce the transcriptional activity of the NF-κB pathway by blocking TNF-α-induced phosphorylation of NF-κB and inhibitor of NF-κB (IκB) and inhibiting IκB degradation [65]. Insulin-like growth factor-1 (IGF-1) and platelet-derived growth factor-BB (PDGF-BB) can also inhibit IL-1β-induced activation of NF-κB and apoptosis of chondrocytes by restraining the Srcc/PI-3 K/AKT pathway and exert anabolic and anti-inflammatory effects in chondrocytes in vitro [66].

Growth factors have been shown to stimulate chondrocyte proliferation and cartilage regeneration and repair. PRP can facilitate the synthesis of chondrocytes and cartilage matrix-related proteins [67], including transforming growth factor β (TGF-β), which was believed to regulate the synthesis of collagen and proteoglycans, thereby accelerating the differentiation and proliferation of chondrocytes and regulating the release of other growth factors [68]. TGF-β1 has been injected into the joint of the TMJOA rabbit model to stimulate the regeneration of articular cartilage and delay its progressive destruction, thus promoting the repair of articular cartilage [69]. In addition, repair of cartilage and subchondral bone in TMJOA was enhanced when IGF-1 was suspended in HA [70].

PRP favors the secretion of endogenous HA by synovial cells [71]. The advantages of HA include restoring synovial fluid viscoelasticity, reducing or eliminating joint friction, improving the maximal mouth opening, relieving pain, and restoring TMJ homeostasis. Considering the underlying mechanism of action of PRP in treating TMD, we consider that PRP can significantly reduce the pain and MMO of the TMJ and is more effective in this regard than HA is. This conclusion agrees with the experimental results of Toameh et al. [50].

Although PRP can effectively improve the symptoms of patients with TMD, antibodies may be produced against clotting factor V, XI, and human thrombin when bovine thrombin is used, which can cause severe coagulopathies [72, 73]. PRP needs additional anticoagulants that may inhibit tissue healing. Furthermore, the tedious preparation for PRP makes this impractical for many outpatients. PRP also releases growth factors for a short time, releasing more in the early stage and less in the later stage, and activation of PRP by thrombin can release growth factors quickly, making the release of cytokines from PRP inconsistent [74].

As the second-generation platelet concentrate, PRF has several merits over PRP. Exogenous additives are not required in the preparation of PRF, which can effectively avoid the adverse reactions of patients, and the preparation technology of PRF is simple and can be obtained via a one-step centrifugation [75]. Moreover, PRF has natural and slow polymerization characteristics in centrifugation. Natural polymerization without thrombin ensures that PRF has a finer and more flexible fibrin network, supporting the release of cytokines and cell migration [75]. The slow fibrin polymerization of PRF contributes to the intrinsic incorporation of platelet cytokines and sugar chains into the fibrin network, which gradually releases cytokines during fibrin matrix remodeling, and this structure is conducive to the healing process [75, 76]. Further studies have shown that injectable PRF (I-PRF) acquired via low-speed centrifugation can continuously release more inflammatory cells, platelets, and growth factors [77]. In addition, the long-term release of higher levels of growth factors can more stimulate cartilage regeneration than PRP can [78, 79]. The mechanism of PRF for treating TMD may be via joint regulation of platelet-release mediators and fibrin matrix. The role of platelet-release mediators in PRF is similar to that in PRP and can inhibit inflammation and promote chondrocyte proliferation and cartilage repair. The fibrin matrix has a unique three-dimensional reticular structure that can support cell migration and enables PRF to slowly release growth factors and cytokines, thus prolonging the action time of factors and contributing to treatment effect. I-PRF is currently the only form of PRF used to treat TMD. Albilia et al. [13] proved that I-PRF stores growth factors and cells in the joint space to ensure long-term release for improvement of TMJ functional activity and pain relief; this state was considered to last for at least 12 months, thus restoring intra-articular homeostasis. Currently, the lack of a unified standard for preparing PRP may produce changes in PRP composition and therapeutic efficacy. According to different preparation methods, leukocytes may be present in PRP. The clinical results and cellular effects of leukocyte-rich or-poor PRP are still controversial [80]. Compared with the more problematic PRP, the composition and preparation of I-PRF tends to be mature (Table 3). At present, PRF has more advantages and appears be a better choice, whereas further research is required to prove the long-term efficacy of PRF.

**Table 3:** Characteristics and differences between PRP and I-PRF

Comparative items	Centrifugal method	Activator	Anticoagulant	Centrifugal components	Release GFs
interventions					
**PRP**	No unified standard	Yes	Yes	PLT; WBCs(with/without); Fibrin(weak)	Rapid
**I-PRF**	700 rpm 3 min	No	No	PLT; WBCs; Fibrin(strong)	Slow

*PRP* Platelet-rich plasma, *I-PRF* Injectable platelet-rich fibrin, *PLT* Platelets, *WBCs* White blood cells, *GFs* Growth factors

## Strengths and limitations of this study

This study compares the efficacy of HA, PRP, and PRF in treating TMD in a network meta-analysis for the first time. The network meta-analysis was performed using the Cochrane collaboration and GRADE system recommendations. Additionally, these interventions were ranked using SUCRA ratings of outcomes, and the best interventions were identified.

Nevertheless, we should interpret the results of the network meta-analysis cautiously because of several limitations in this study. First, TMD has a multifactorial etiology. Network meta-analysis results may be affected by the difference in diagnosis between patients in each study. Second, according to the GRADE system, our network results contained imprecision and inconsistency. The differences in the preparation scheme and dosage of PRP presumably generate differences in the effect of PRP, which affected some comparisons between studies. Furthermore, the centrifugal parameters and dose used in several studies were not reported, leading to subgroup analysis difficulties, and a small sample size also contributes to the difference in results despite a relatively low bias risk.

## Conclusion

This study compared the efficacy of HA, PRP, and PRF in treating TMD. PRF appears to be more effective at relieving pain and improving MMO in patients with TMD. However, more studies are required to fully determine the efficacy of this treatment.

### Supplementary Information

**Additional file 1.** Search strategy.

**Additional file 2.** The forest plot. (A) The forest plot about pain after one monthtreatment. (B) The forest plot about pain after three months treatment. (C) Theforest plot about pain after six months treatment. (D) The forest plot aboutMMO after one month treatment. (E) The forest plot about MMO after three monthstreatment. (F) The forest plot about MMO after six months treatment. HA:hyaluronic acid; PRP: platelet-rich plasma; PRF: platelet-rich fibrin; PO:placebo; MMO: maximal mouth opening.

**Additional file 3.** The funnel plot. (A) The funnel plot about pain after one month,three months and six months treatment. (B) The funnel plot about MMO after onemonth, three months and six months treatment. MMO: maximal mouth opening.

## Data Availability

The datasets used in this study are available from the corresponding authors upon reasonable request.

## Abbreviations

HA: Hyaluronic acid
PRP: Platelet-rich plasma
PRF: Platelet-rich fibrin
PO: Placebo
TMD: Temporomandibular disorders
MMO: Maximal mouth opening
VAS: Visual analog scale
GRADE: Grade of Recommendations AssessmentDevelopment and Evaluation
RCTs: Randomized controlled trials
ID: Internal derangements
DDwR: Disc displacement with reduction
DDwoR: Disc displacement without reduction
I-PRF: Injectable platelet-rich fibrin
PLT: Platelets
WBCs: White blood cells
GFs: Growth factors
