# Supplementary material for: Comparative effectiveness of hyaluronic acid, platelet-rich plasma, and platelet-rich fibrin in treating temporomandibular disorders: a systematic review and network meta-analysis
Source: Head Face Med. 2023 Aug 26;19:39. doi: 10.1186/s13005-023-00369-y (PMC10463486; doi:10.1186/s13005-023-00369-y)
Supplement: Supplementary file 1 — Additional file 1. Search strategy. [file 13005_2023_369_MOESM1_ESM.pdf]

Additional file 1 Search strategy

**PubMed**	
#1	"temporomandibular joint disorders"[MeSH Terms]
#2	"temporomandibular joint disorder"[Title/Abstract] OR "temporomandibular joint disorders"[Title/Abstract] OR "temporomandibular joint disease"[Title/Abstract] OR "temporomandibular joint diseases"[Title/Abstract] OR "temporomandibular disorder"[Title/Abstract] OR "temporomandibular disorders"[Title/Abstract] OR "tmj disorder"[Title/Abstract] OR "tmj disorders"[Title/Abstract] OR "tmj disease"[Title/Abstract] OR "tmj diseases"[Title/Abstract] OR "temporomandibular joint dysfunction syndrome"[Title/Abstract] OR "disc displacement"[Title/Abstract] OR "internal derangement"[Title/Abstract] OR "joint dislocations"[Title/Abstract]
#3	#1 OR #2
#4	"platelet-rich plasma"[MeSH Terms]
#5	"platelet-rich plasma"[Title/Abstract] OR "blood platelets"[Title/Abstract] OR "Plasma"[Title/Abstract] OR ("Fluids"[Title/Abstract] AND "Secretions"[Title/Abstract]) OR "body fluids"[Title/Abstract] OR "Blood"[Title/Abstract] OR "platelet-rich fibrin"[Title/Abstract] OR "extracellular fluid"[Title/Abstract] OR ("Hemic"[Title/Abstract] AND "immune systems"[Title/Abstract]) OR "platelet-rich plasma"[Title/Abstract]
#6	#4 OR #5
#7	"Hyaluronic Acid"[MeSH Terms]
#8	"Hyaluronic Acid"[Title/Abstract] OR "Hyaluronan"[Title/Abstract] OR "Hyaluronate"[Title/Abstract]
#9	#7 OR #8
#10	#6 OR #9
#11	"randomized controlled trial"[Publication Type] OR "controlled clinical trial"[Publication Type] OR "Randomized"[Title/Abstract] OR "placebo"[Title/Abstract] OR "randomly"[Title/Abstract] OR "Trial"[Title/Abstract] OR "groups"[Title/Abstract]
#12	#3 AND #10 AND #11
**Embase**	
#1	'temporomandibular joint disorder'/exp
#2	'temporomandibular joint disease':ti,ab OR 'temporomandibular joint diseases':ti,ab OR 'temporomandibular joint disorder':ti,ab OR 'temporomandibular joint disorders':ti,ab OR 'temporomandibular joint dysfunction syndrome':ti,ab OR 'temporomandibular disorder':ti,ab OR 'temporomandibular disorders':ti,ab OR 'tmj disorder':ti,ab OR 'tmj disorders':ti,ab OR 'tmj disease':ti,ab OR 'tmj diseases':ti,ab OR 'disc displacement':ti,ab OR 'internal derangement':ti,ab OR 'joint dislocations':ti,ab
#3	#1 OR #2
#4	'platelet-rich plasma'/exp
#5	'platelet-rich plasma':ti,ab OR 'platelet rich plasma':ti,ab OR 'blood platelets':ti,ab OR plasma:ti,ab OR 'fluids and secretions':ti,ab OR 'body fluids':ti,ab OR blood:ti,ab OR 'platelet-rich fibrin':ti,ab OR 'extracellular fluid':ti,ab OR hemic:ti,ab OR 'immune systems':ti,ab

#6	#4 OR #5
#7	'hyaluronic acid'/exp
#8	'hyaluronic acid':ti,ab OR 'hyaluronan':ti,ab OR 'hyaluronate':ti,ab
#9	#7 OR #8
#10	#6 OR #9
#11	'randomized controlled trial':ti OR 'controlled clinical trial':ti OR randomized:ti,ab OR placebo:ti,ab OR randomly:ti,ab OR trial:ti,ab OR groups:ti,ab
#12	#3 AND #10 AND #11
**Cochrane Library**	
#1	MeSH descriptor: [Temporomandibular Joint Disorders] explode all trees
#2	(Temporomandibular Joint Disorder):ti,ab,kw OR (Temporomandibular Joint Disease):ti,ab,kw OR (Temporomandibular Disorder):ti,ab,kw OR (TMJ Disorder):ti,ab,kw OR (TMJ Disease):ti,ab,kw OR (Temporomandibular Joint Dysfunction Syndrome):ti,ab,kw OR (Disc Displacement):ti,ab,kw OR (Internal Derangement):ti,ab,kw OR (Joint Dislocations):ti,ab,kw
#3	#1 OR #2
#4	MeSH descriptor: [Platelet-Rich Plasma] explode all trees
#5	(platelet-rich plasma):ti,ab,kw OR (blood platelets):ti,ab,kw OR (plasma):ti,ab,kw OR (fluids and secretions):ti,ab,kw OR (body fluids):ti,ab,kw OR (blood):ti,ab,kw OR (platelet-rich fibrin):ti,ab,kw OR (extracellular fluid):ti,ab,kw OR (hemic and immune systems):ti,ab,kw OR (platelet rich plasma):ti,ab,kw
#6	#4 OR #5
#7	MeSH descriptor: [Hyaluronic Acid] explode all trees
#8	(hyaluronic acid):ti,ab,kw OR (hyaluronan):ti,ab,kw OR (hyaluronate):ti,ab,kw
#9	#7 OR #8
#10	#6 OR #9
#11	(randomized controlled trial):pt OR (controlled clinical trial):pt OR (randomized):ti,ab,kw OR (placebo):ti,ab,kw OR (randomly):ti,ab,kw OR (trial):ti,ab,kw OR (groups):ti,ab,kw
#12	#3 AND #10 AND #11
**Scopus**	
#1	INDEXTERMS (temporomandibular AND joint AND disorders)
#2	TITLE-ABS-KEY-AUTH (temporomandibular AND joint AND disorder) OR TITLE-ABS-KEY (temporomandibular AND joint AND disorders) OR TITLE-ABS-KEY (temporomandibular AND joint AND disease) OR TITLE-ABS-KEY (temporomandibular AND joint AND diseases) OR TITLE-ABS-KEY (temporomandibular AND disorder) OR TITLE-ABS-KEY (temporomandibular AND disorders) OR TITLE-ABS-KEY (tmj AND disorder) OR TITLE-ABS-KEY (tmj AND disorders) OR TITLE-ABS-KEY (tmj AND disease) OR TITLE-ABS-KEY (tmj AND diseases) OR TITLE-ABS-KEY (temporomandibular AND joint AND dysfunction AND syndrome) OR TITLE-ABS-KEY (disc AND displacement) OR TITLE-ABS-KEY (internal AND derangement) OR TITLE-ABS-KEY (joint AND dislocations)
#3	#1 OR #2
#4	INDEXTERMS (platelet-rich AND plasma)
#5	TITLE-ABS-KEY-AUTH (platelet-rich AND plasma) OR TITLE-ABS-KEY (blood AND pl

	atelets) OR TITLE-ABS-KEY (plasma) OR TITLE-ABS-KEY (fluids AND secretions) OR TITLE-ABS-KEY (body AND fluids) OR TITLE-ABS-KEY (blood) OR TITLE-ABS-KEY (platelet-rich AND fibrin) OR TITLE-ABS-KEY (extracellular AND fluid) OR TITLE-ABS-KEY (hemic AND immune AND systems) OR TITLE-ABS-KEY (platelet AND rich AND plasma)
#6	#4 OR #5
#7	INDEXTERMS (hyaluronic AND acid)
#8	TITLE-ABS-KEY-AUTH (hyaluronic AND acid) OR TITLE-ABS-KEY (hyaluronan) OR TITLE-ABS-KEY (hyaluronate)
#9	#7 OR #8
#10	#6 OR #9
#11	TITLE-ABS-KEY (randomized AND controlled AND trial) OR TITLE-ABS-KEY (controlled AND clinical AND trial) OR TITLE-ABS-KEY (randomized) OR TITLE-ABS-KEY (placebo) OR TITLE-ABS-KEY (randomly) OR TITLE-ABS-KEY (trial) OR TITLE-ABS-KEY (groups)
#12	#3 AND #10 AND #11
**Web of science**	
#1	TS=(Temporomandibular Joint Disorders)
#2	TI=(Temporomandibular Joint Disorder OR Temporomandibular Joint Disorders OR Temporomandibular Joint Disease OR Temporomandibular Joint Diseases OR Temporomandibular Disorder OR Temporomandibular Disorders OR TMJ Disorder OR TMJ Disorders OR TMJ Disease OR TMJ Diseases OR Temporomandibular Joint Dysfunction Syndrome)
#3	AB=(Temporomandibular Joint Disorder OR Temporomandibular Joint Disorders OR Temporomandibular Joint Disease OR Temporomandibular Joint Diseases OR Temporomandibular Disorder OR Temporomandibular Disorders OR TMJ Disorder OR TMJ Disorders OR TMJ Disease OR TMJ Diseases OR Temporomandibular Joint Dysfunction Syndrome)
#4	AK=(Temporomandibular Joint Disorder OR Temporomandibular Joint Disorders OR Temporomandibular Joint Disease OR Temporomandibular Joint Diseases OR Temporomandibular Disorder OR Temporomandibular Disorders OR TMJ Disorder OR TMJ Disorders OR TMJ Disease OR TMJ Diseases OR Temporomandibular Joint Dysfunction Syndrome)
#5	#1 OR #2 OR #3 OR #4
#6	TS=(platelet-rich plasma)
#7	TI=(platelet-rich plasma OR platelet rich plasma OR blood platelets OR plasma OR fluids and secretions OR body fluids OR blood OR platelet-rich fibrin OR extracellular fluid OR hemic OR immune systems)
#8	AB=(platelet-rich plasma OR platelet rich plasma OR blood platelets OR plasma OR fluids and secretions OR body fluids OR blood OR platelet-rich fibrin OR extracellular fluid OR hemic OR immune systems)
#9	AK=(platelet-rich plasma OR platelet rich plasma OR blood platelets OR plasma OR fluids and secretions OR body fluids OR blood OR platelet-rich fibrin OR extracellular fluid OR

	hemic OR immune systems)
#10	#6 OR #7 OR #8 OR #9
#11	TS=(hyaluronic acid)
#12	TI=(hyaluronic acid OR hyaluronan OR hyaluronate)
#13	AB=(hyaluronic acid OR hyaluronan OR hyaluronate)
#14	AK=(hyaluronic acid OR hyaluronan OR hyaluronate)
#15	#11 OR #12 OR #13 OR #14
#16	#10 OR #15
#17	TI=(randomized controlled trial OR controlled clinical trial OR randomized OR placebo OR randomly OR trial OR groups)
#18	AB=(randomized controlled trial OR controlled clinical trial OR randomized OR placebo OR randomly OR trial OR groups)
#19	#17 OR #18
#20	#5 AND #16 AND #19
