# Supplementary material for: Comparative effectiveness of hyaluronic acid, platelet-rich plasma, and platelet-rich fibrin in treating temporomandibular disorders: a systematic review and network meta-analysis
Source: Head Face Med. 2023 Aug 26;19:39. doi: 10.1186/s13005-023-00369-y (PMC10463486; doi:10.1186/s13005-023-00369-y)
Supplement: Supplementary file 2 — Additional file 2. The forest plot. (A) The forest plot about pain after one monthtreatment. (B) The forest plot about pain after three months treatment. (C) Theforest plot about pain after six months treatment. (D) The forest plot aboutMMO after one month treatment. (E) The forest plot about MMO after three monthstreatment. (F) The forest plot about MMO after six months treatment. HA:hyaluronic acid; PRP: platelet-rich plasma; PRF: platelet-rich fibrin; PO:placebo; MMO: maximal mouth opening. [file 13005_2023_369_MOESM2_ESM.pdf]

Additional file 2

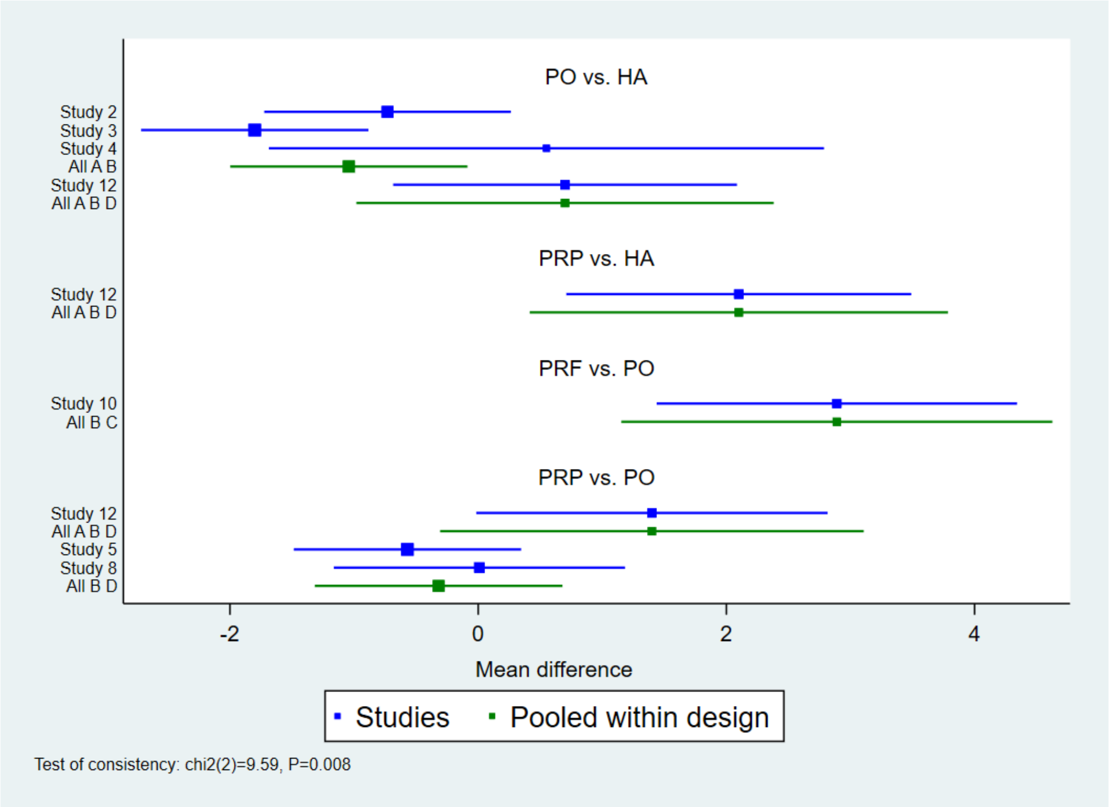

(A) The forest plot about pain after one month treatment.

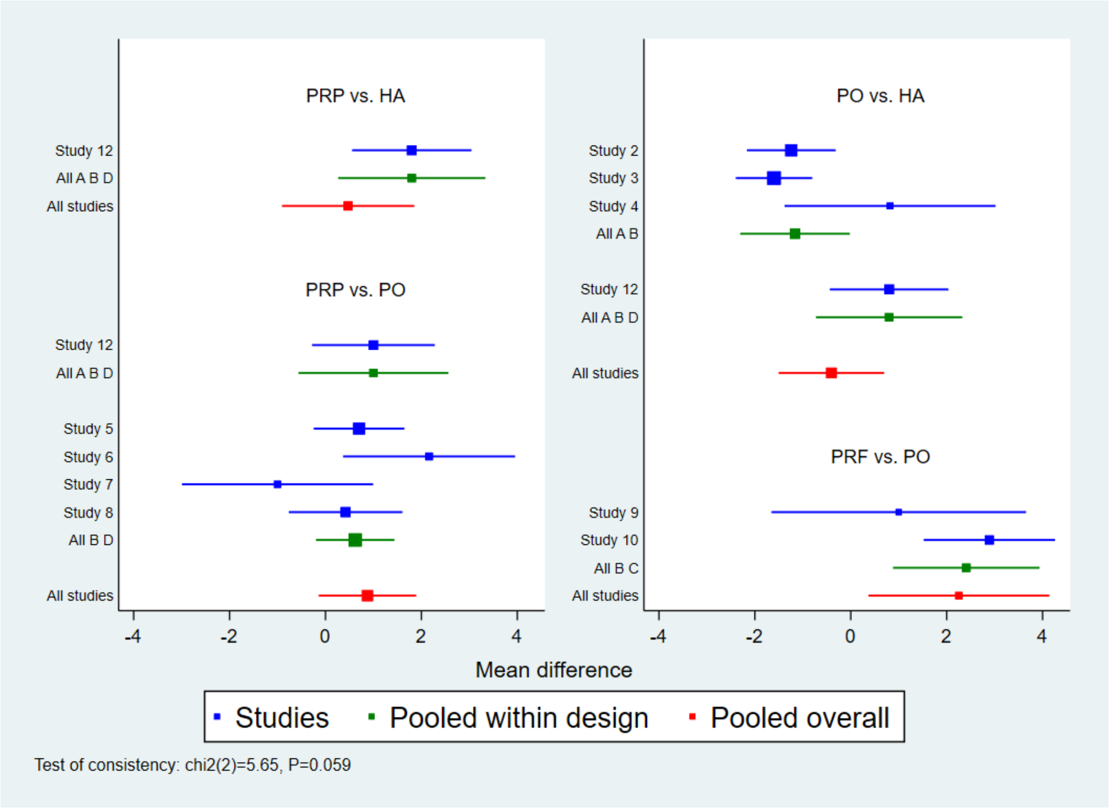

(B) The forest plot about pain after three months treatment.

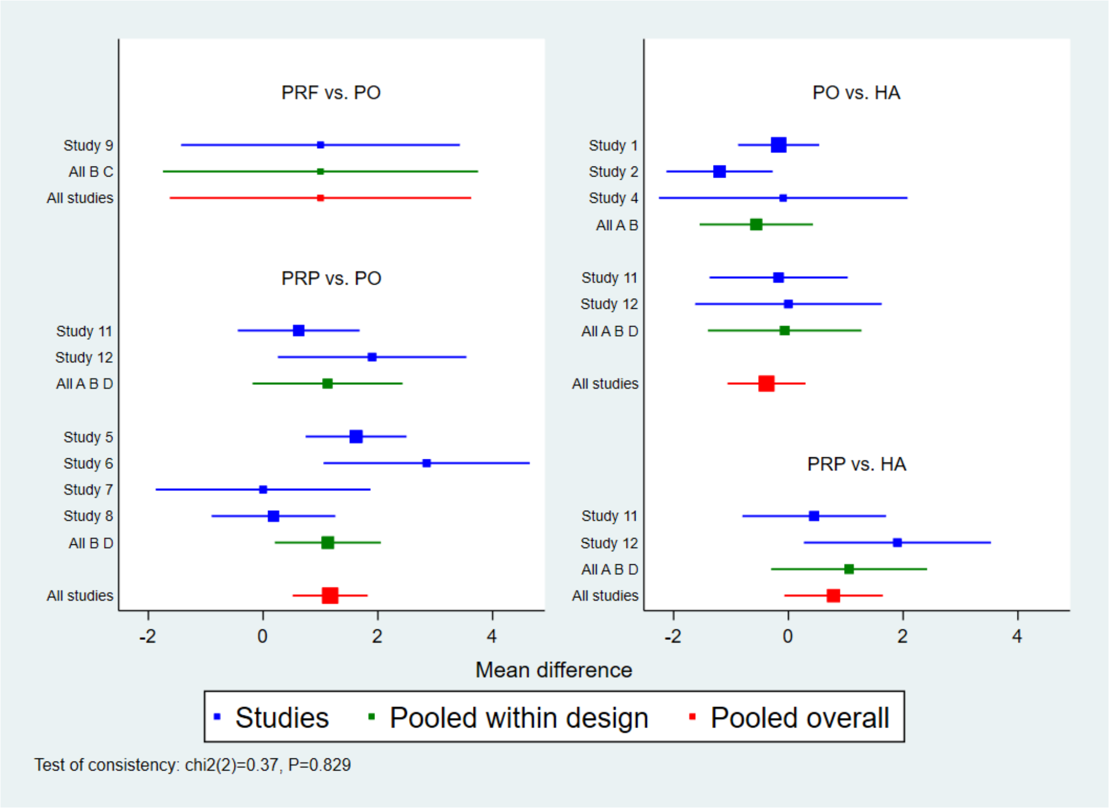

(C) The forest plot about pain after six months treatment.

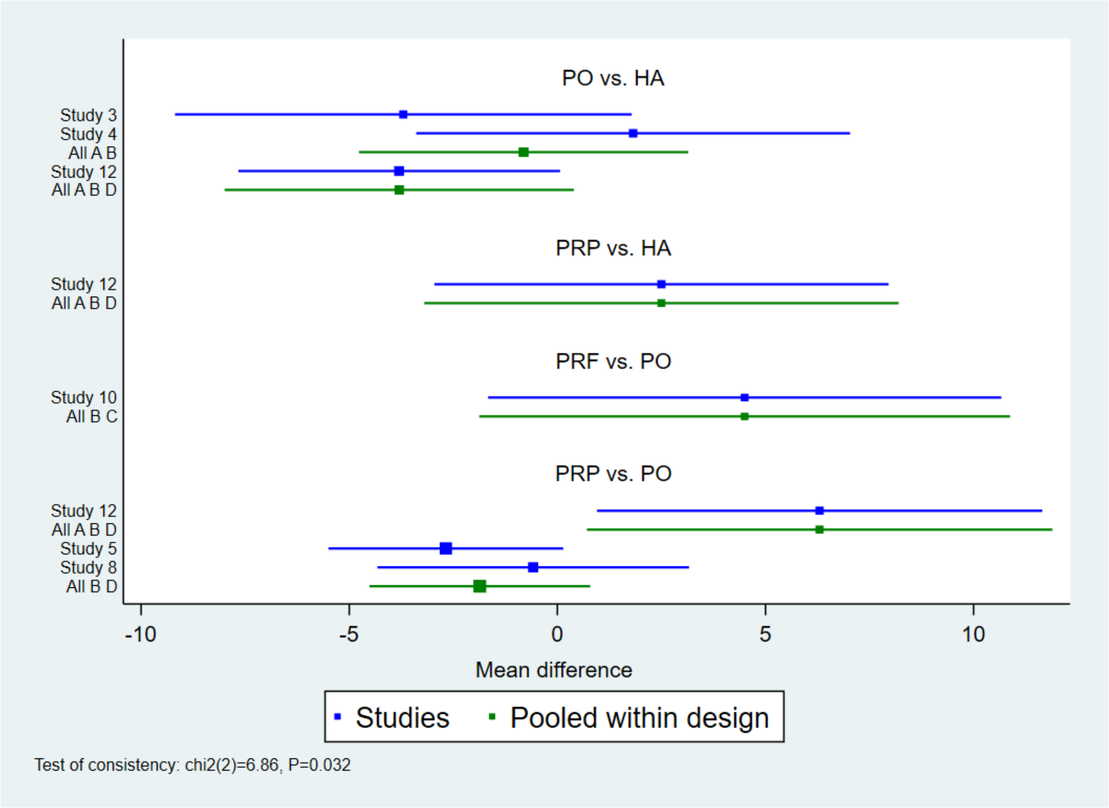

(D) The forest plot about MMO after one month treatment. MMO: maximum mouth opening.

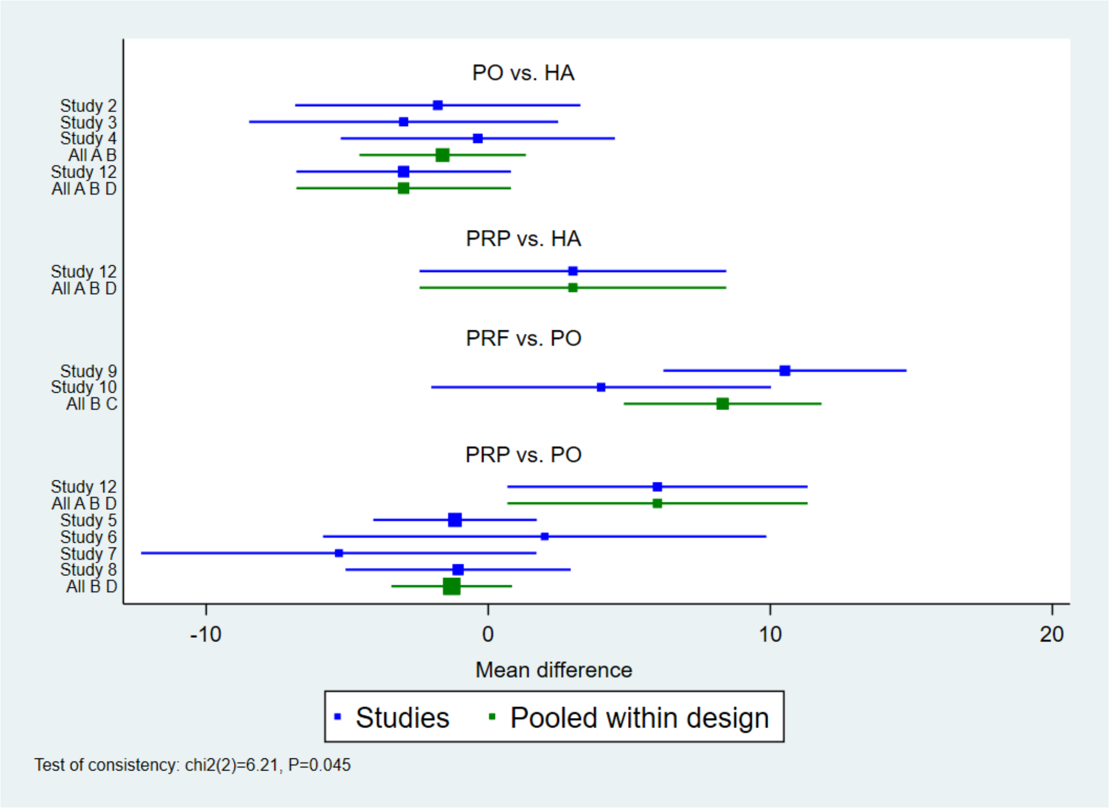

(E) The forest plot about MMO after three months treatment. MMO: maximum mouth opening.

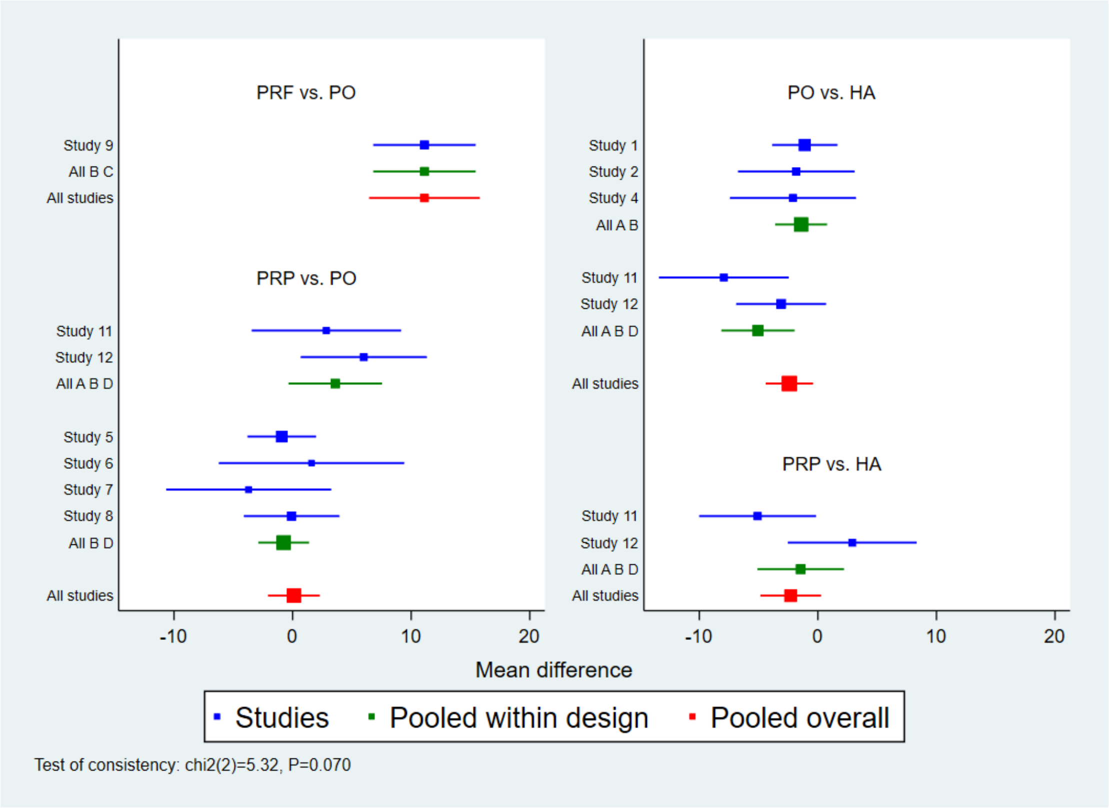

(F) The forest plot about MMO after six months treatment. MMO: maximum mouth opening.
