# Supplementary material for: Comparative effectiveness of hyaluronic acid, platelet-rich plasma, and platelet-rich fibrin in treating temporomandibular disorders: a systematic review and network meta-analysis
Source: Head Face Med. 2023 Aug 26;19:39. doi: 10.1186/s13005-023-00369-y (PMC10463486; doi:10.1186/s13005-023-00369-y)
Supplement: Supplementary file 3 — Additional file 3. The funnel plot. (A) The funnel plot about pain after one month,three months and six months treatment. (B) The funnel plot about MMO after onemonth, three months and six months treatment. MMO: maximal mouth opening. [file 13005_2023_369_MOESM3_ESM.pdf]

Additional file 3

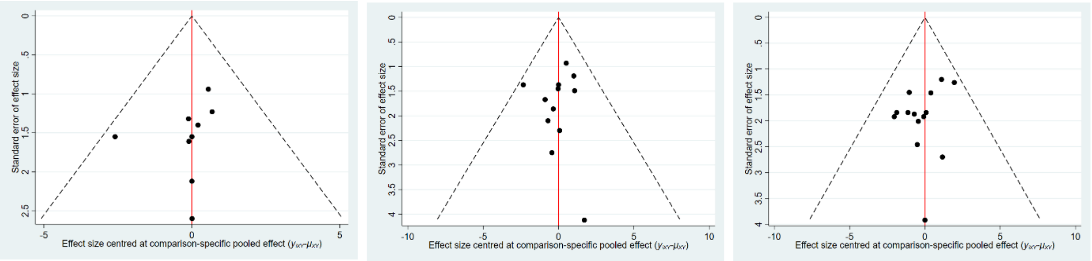

(A) The funnel plot about pain after one month, three months and six months treatment.

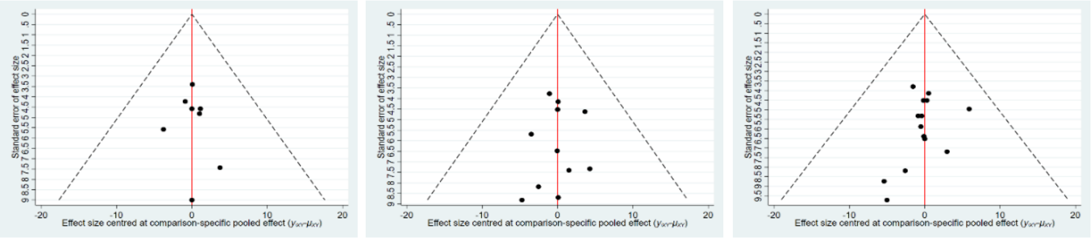

(B) The funnel plot about MMO after one month, three months and six months treatment. MMO: maximum mouth opening.
